# Editorial: Iron Metabolism at the Crossroad of Innate Immune Response and Cancer Progression

**DOI:** 10.3389/fimmu.2021.832886

**Published:** 2022-01-05

**Authors:** Paola Zacchi, Stefania Recalcati

**Affiliations:** ^1^ Department of Life Sciences, University of Trieste, Trieste, Italy; ^2^ Department of Biomedical Sciences for Health , University of Milan, Milano, Italy

**Keywords:** iron metabolism, cancer biology, innate immunity, macrophages, ferroptosis

Iron is a vital nutrient indispensable for the activity of many proteins and enzymes sustaining essential cellular processes such as oxygen transport, energy production, DNA synthesis and repair, cell growth and death, detoxification and host defence (1). Given its high redox activity iron can cause cellular toxicity upon reactive oxygen species (ROS) production *via* Fenton chemistry, being potentially mutagenic (2). Therefore, systemic and cellular iron availability must be tightly regulated.

Iron metabolism is often deregulated in malignant cells, due to their higher metabolic demand cancer cells develop to fuel proliferation, survival, motility and invasion (reviewed in 3). To this goal cancer cells not only enhance their iron import pathways while reducing export, but they also affect how iron is handled by innate and adaptive immune cells, such as macrophages, neutrophils and T cells (4). The innate immune cells are responsible for initiating inflammatory responses aimed at defending body against pathogens but also malignant cells. Therefore, immune cells sequester iron in order to subtract it from tumor cells availability (5). If the inflammatory stimulus persists and becomes chronic, a dangerous interplay between cancer cells and infiltrating leukocytes is established, leading to a drastic change in their polarization toward an immunosuppressive and tumor-supportive phenotype (6). These changes are intimately associated with alterations in iron handling at both tumor and systemic levels, and macrophages, the sentinels of the innate immunity, can be considered the master regulator of all these complex and interconnected events (7).

In this Research Topic, two reviews manuscripts explore the multifaceted role macrophages play in promoting or hampering tumor development based on their ability to influence and be influenced by cancer cells, and how these reciprocal interactions shape iron metabolism.

Liang and Ferrara illustrate extensively the different mechanisms macrophages residing in the tumor microenvironment (TME) put in place, instructed by cancer cells, to become sources of iron and iron-related proteins requested for tumor outgrowth and the signalling pathways involved in these processes. They also include the contribution of neutrophils as iron-donor component of the TME. Neutrophils infiltrating cancerous lesions have long been considered a mere bystander since it was hard to believe that such short-lived leukocytes could perform relevant roles on a chronic and progressive disease like cancer (8). Many recent studies have challenged this view, demonstrating that neutrophils are extremely plastic and can undergo “alternative activation” upon exposure to various cues found in the TME, driving either anti-tumor or pro-tumor functions (9). Therefore, their contribution in cancer development and progression cannot be neglected, neither their active involvement in iron dysregulation.

Another review by DeRosa and Leftin examines how tumor-associated macrophages decline systemic iron metabolism alterations with changes in the iron content of the TME and how these changes, shaping their iron polarization phenotypes, impact on the efficacy of the immune response against cancer. They describe as “iron curtain” the characteristic distribution of iron-loaded TAMs that creates a physical border at the tumor front, which would allow TAMs to exert control over metabolic flux and immune response. There are still several issues that need further investigation. It is still poorly understood how spatial heterogeneity in TME affects the way iron is exchanged between cancer cells and macrophages, alters macrophage communication with other tumor associated cell types and how systemic iron dynamics impact on macrophage plasticity during the different steps of cancer progression. These questions need to be carefully addressed to design new anti-cancer therapies targeting the immune-metabolic axis.

Then Weiler and Nairz, in their Hypothesis and Theory contribution, comprehensively address the multifactorial pathophysiology of cancer induced anaemia (CIA). Mechanistically, CIA represents a cytokine-mediated disorder arising from the complex communication established between cancer cells and the immune system. Again, macrophages are of critical importance since their activation makes them source of many proinflammatory cytokines (e.g., IFN-γ, TNF-α, IL-1, and IL-6) that lead to insufficient erythropoiesis mainly through two mechanisms: iron restriction and functional impairment of erythropoietic progenitors. In most cases, this condition negatively impacts on the efficacy of anti-cancer treatments and therefore on patient survival. Several strategies can be adopted to ameliorate CIA, such as hepcidin antagonism, iron supplementation and erythropoiesis-stimulating agents (ESA), but the unintended effects that CIA-directed therapies may exert on TAMs in particular, and other tumor infiltrating leukocytes, are still poorly characterized. Further mechanistic insights need to be provided in order to unveil secondary effects of CIA treatments that may negatively impact on the course of the disease.

In the continuing theme Tymoszuk et al. demonstrate that intravenous iron supplements for curing CIA significantly hampered the T-cell mediated immune response against a murine implanted mammary carcinomas cancer. Key effector cells in antitumor immunity are cytotoxic CD8+ T cells and of CD4+ T helper cells type 1, these latter sustaining the activation, expansion and cell killing activities of the CD8+ T cells (10). This correlates with the fact that most of tumor neo-antigens arising from somatic mutations are presented by the major histocompatibility complex (MHC) class I (11). The authors showed that iron supply has detrimental effects CD8+ T cells proliferation, cytokines production and degranulation. Several mechanisms could be involved, such as ROS-dependent cell death of tumor infiltrating lymphocytes, iron-dependent impairment of T cell receptor signalling or damping of co-stimulatory pathways. Therefore, iron supplementation in cancer patients should be carefully pondered, especially in those treated with immunotherapies.

Weber et al., in their review article, tackle another important aspect of iron dysregulation in patients affected by Myelodysplastic syndromes (MDS), a heterogeneous group of myeloid neoplasms characterized by inefficient hematopoiesis and a risk of progression to acute myeloid leukemia (AML). Anemia and thrombocytopenia are common presenting features in these affected patients and transfusion supportive care is the therapeutic option usually applied to ameliorate the quality of life. Unfortunately, this treatment promotes a secondary iron overload in the bone marrow, while the first arising from disease-dependent insufficient erythropoiesis. This condition is accompanied by ROS production which may contribute to leukemogenesis. In the extreme, iron dependent overwhelming accumulation of ROS could be exploited to promote cell death *via* a novel form of regulated cell death strictly dependent on iron metabolism called ferroptosis (12). This process is driven by the lethal accumulation of lipid peroxidation (13), and multiple genes have been identified as modulators, drivers or markers for this type of iron-dependent cell death in diverse types of cancer.

In Hepatocellular carcinoma (HCC), a highly aggressive cancer with limited therapeutic interventions, accumulating evidence unveiled that ferroptosis performs a key role in regulating the development and progression of this malignancy, the immune status and the anti-tumor response (14). The paper of Liu et al. identified and validated two heterogenous ferroptosis subtypes: the first one was characterized by low expression of Ferroportin releated genes (FRGs) and high load of innate and adaptive cells, vice-versa the second group has an opposite phenotype. Based on FRGs expression, cells infiltration, immune escape mechanisms, genome-driven events and clinical outcomes of the two ferroptosis subtypes, they proposed a scoring system termed ferroptosis related risk score (FRRS), which is expected to reliably assess prognosis and to improve the clinical management of HCC.

The review by Aksan et al. focuses on another aspect of iron genotoxic potential, mostly attributed and studied under iron overload conditions, but found tumorigenic also under iron deficiency context. This is the case of the colorectal cancer, whose pathogenesis has been linked to reduced iron intake and low systemic iron levels. Being iron an essential cofactor for the full performance of a wide variety of enzymes involved in DNA replication and repair, microRNA biogenesis and anti-oxidant systems, its insufficient supply is expected to impair cell mediated immunity and immunosurveillance, activities strictly dependent on iron status (15). Therefore, iron deficiency as well as iron surplus can be considered two sides of the same coin, both negatively impacting on tumorigenesis, cancer progression and clinical outcomes.

The last two papers, from Fan et al. and Zacchi et al. deal with the function and clinical significance of the expression of two key players of cellular iron export in lung cancer pathogenesis and prognosis, namely hepcidin and hephaestin (HEPH). As previously mentioned, hepatic hepcidin regulates systemic iron availability by suppressing intestinal iron absorption and iron egress from macrophages upon down-regulation of the only known mammalian iron exporter ferroportin (16). Hepcidin synthesis can also occur at extrahepatic location upon diverse stimuli such as iron excess, hypoxia and inflammatory cytokines and cancer cells can locally produce it to sustain their iron-utilization phenotype (17). Even though the functional significance of this local hepcidin production is still poorly understood, Fan et al., based on mRNA expression dataset, found a positive correlation between hepcidin expression and the infiltration levels of lymphocytic cells, neutrophils, macrophages, and dendritic cells. Since patients with high hepcidin expression exhibited a markedly worse survival rate than those with low expression, this may render hepcidin a novel immune-related actor in lung cancer and an independent prognostic biomarker.

In the same cancer context, Zacchi et al., by means of bioinformatics, studied the expression and prognostic value of HEPH, a ferroxidase functionally coupled with ferroportin, that promotes iron export *via* ferrous iron oxidation into its ferric form. HEPH emerged to reside mostly on stromal cellular elements, such as endothelial cells and fibroblasts, key players of the tumorigenic process. Upregulation of HEPH expression correlates with a better outcome as low expression of hepcidin, since both conditions decrease intracellular free iron concentration, known to boost cell proliferation.

In summary, in this Research Topic, leading scientists provided a current state of the art on the role of iron metabolism as a player connecting cancer and immune cells, and its contribution to tumor progression.

## Author Contributions

All authors listed have made a substantial and intellectual contribution to the work, and approved it for publication.

## Conflict of Interest

The authors declare that the research was conducted in the absence of any commercial or financial relationships that could be construed as a potential conflict of interest.

## Publisher’s Note

All claims expressed in this article are solely those of the authors and do not necessarily represent those of their affiliated organizations, or those of the publisher, the editors and the reviewers. Any product that may be evaluated in this article, or claim that may be made by its manufacturer, is not guaranteed or endorsed by the publisher.
